# 

**Published:** 1890-02-22

**Authors:** 


PRICE ONE PENNY.

No. 178, Vol. VII.—February 22nd, 1890.

[Registered at the General Post Office as a Newspaper for transmission in the United Kingdom and Abroad.

WITH EXTRA NURSING SUPPLEMENT.
Per Annum, 6s. 6d., post free.
Monthly Parts, 6d.; post free 8d.
Ditto, per Annum, 8s., post free.

### THE HOSPITAL

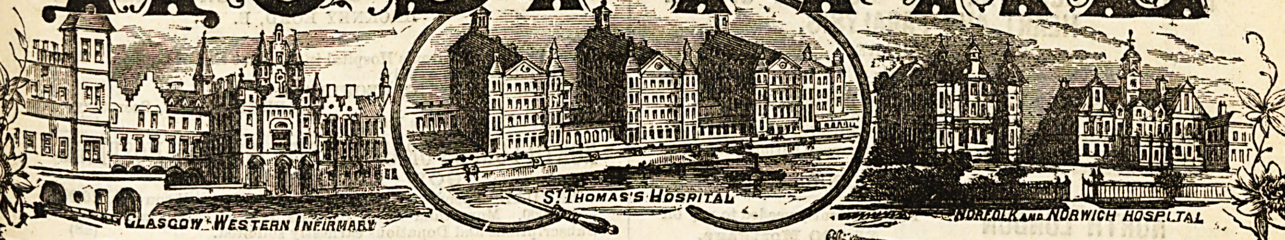

A WEEKLY JOURNAL OF

Science, Medicine, Nursing and Philanthropy.

SATURDAY, FEBRUARY 22nd, 1890.

